# Identify RNA-associated subcellular localizations based on multi-label learning using Chou’s 5-steps rule

**DOI:** 10.1186/s12864-020-07347-7

**Published:** 2021-01-15

**Authors:** Hao Wang, Yijie Ding, Jijun Tang, Quan Zou, Fei Guo

**Affiliations:** 1 School of Computer Science and Technology, College of Intelligence and Computing, Tianjin University, Tianjin, China; 2 School of Electronic and Information Engineering, Suzhou University of Science and Technology, Suzhou, China; 3 Institute of Fundamental and Frontier Sciences, University of Electronic Science and Technology of China, Chengdu, Sichuan China; 4 School of Computational Science and Engineering, University of South Carolina, Columbia, 29208 SC US

**Keywords:** RNA subcellular localization, Multi-label classification, Hilbert-Schmidt independence criterion, Multiple kernel learning, Web server

## Abstract

**Background:**

Biological functions of biomolecules rely on the cellular compartments where they are located in cells. Importantly, RNAs are assigned in specific locations of a cell, enabling the cell to implement diverse biochemical processes in the way of concurrency. However, lots of existing RNA subcellular localization classifiers only solve the problem of single-label classification. It is of great practical significance to expand RNA subcellular localization into multi-label classification problem.

**Results:**

In this study, we extract multi-label classification datasets about RNA-associated subcellular localizations on various types of RNAs, and then construct subcellular localization datasets on four RNA categories. In order to study Homo sapiens, we further establish human RNA subcellular localization datasets. Furthermore, we utilize different nucleotide property composition models to extract effective features to adequately represent the important information of nucleotide sequences. In the most critical part, we achieve a major challenge that is to fuse the multivariate information through multiple kernel learning based on Hilbert-Schmidt independence criterion. The optimal combined kernel can be put into an integration support vector machine model for identifying multi-label RNA subcellular localizations. Our method obtained excellent results of 0.703, 0.757, 0.787, and 0.800, respectively on four RNA data sets on average precision.

**Conclusion:**

To be specific, our novel method performs outstanding rather than other prediction tools on novel benchmark datasets. Moreover, we establish user-friendly web server with the implementation of our method.

**Supplementary Information:**

The online version contains supplementary material available at (10.1186/s12864-020-07347-7).

## Background

Biological functions of biomolecules rely on various cellular compartments. One cell can be divided into different compartments that are related to different biological processes. Thus, the cellular role of one RNA molecular could be inferred from its localization information. What’s more, there has been a great deal of research on the protein subcellular localization [1–6]. Currently, the biological technology capable of whole-genome that subcellular localization has been indicated to be a fundamental regulation mode in biological cells [7].

With the explosive growth of biological sequences in the post-genomic era, one of the most important but also most difficult problems in computational biology is how to express a biological sequence with a discrete model or a vector, yet still keep considerable sequence-order information or key pattern characteristic. This is because all the existing machine-learning algorithms, such as Optimization algorithm [8], Covariance Discriminant algorithm [9, 10], Nearest Neighbor algorithm [11], and Support Vector Machine algorithm [11, 12]) can only handle vectors as elaborated in a comprehensive review [13]. However, a vector defined in a discrete model may completely lose all the sequence-pattern information. To avoid completely losing the sequence-pattern information for proteins, the pseudo amino acid composition [14] or PseAAC [15] was proposed. Ever since the concept of Chou’s PseAAC was proposed, it has been widely used in nearly all the areas of computational proteomics [16–18]. Because it has been widely and increasingly used, four powerful open access soft-wares, called ‘PseAAC’ [19], ‘PseAAC-Builder’ [20], ‘propy’ [21], and ‘PseAAC-General’ [22], were established: the former three are for generating various modes of Chou’s special PseAAC [23]; while the 4th one for those of Chou’s general PseAAC[24], including not only all the special modes of feature vectors for proteins but also the higher level feature vectors such as Functional Domain mode, Gene Ontology mode, and Sequential Evolution or Position-Specific Score Matrix(PSSM) mode. Encouraged by the successes of using PseAAC to deal with protein/peptide sequences, the concept of PseKNC (Pseudo K-tuple Nucleotide Composition) [25] was developed for generating various feature vectors for DNA/RNA sequences [26–28] that have proved very useful as well. Particularly, in 2015 a very powerful web-server called Pse-in-One [29] and its updated version Pse-in-One2.0 [30] have been established that can be used to generate any desired feature vectors for protein/peptide and DNA/RNA sequences according to the need of users’ studies. Inspired by the Chou’s method[31, 32], we mainly extract the frequency information of the sequence.

Currently, the biological technology capable of whole-genome localization is the subcellular RNA sequencing, called SubcRNAseq, which yields high-throughput and quantitative data. Large amounts of raw subcRNAseq data have recently become available, most notably from the ENCODE consortium. A lot of research work has established the resource to make RNA localization data available to the broader scientific community. Firstly, Zhang et al. [33] built a database called RNALocate, which collected more than 42,000 manually engineered RNA subcellular localization entries. Subsequently, Mas-Ponte et al. [34] constructed a database named LncATLAS to store the subcellular localization of lncRNA. ViRBase[35] is a resource for studying ncRNA-associated interactions between virus and host. Now, Huang et al.[36] have built a manually curated resource of experimentally supported RNAs with both protein-coding and noncoding function.

Considering expensive and inconvenient biological experiments [37], automatic computational tools are the highly relevant measure to speed up RNA-related studies. The computational identification of subcellular localization has been a hot topic for the last decade. In the early days, Cheng et al. [38] systematically studied the distribution of lncRNA localization in gastric cancer and revealed its relationship with gastric cancer. As a pioneer work, Feng et al. [39] developed a computational method to predict the organelle positions of non-coding RNA (ncRNAs) by collecting ncRNAs from centroids, mitochondria, and chloroplast genomes. Subsequently, Zhen et al. [40] developed lncLocator to predict the subcellular localization of long-stranded non-coding RNA. Xiao et al. [41] proposed a novel method used the sequence-to-sequence model to predict microRNA subcellular localization. Besides, Yang et al. [42] developed MiRGOFS being a GO-based functional similarity measurement for miRNA subcellular localization. Then, iLoc-mRNA [43] used binomial distribution and one-way analysis of variance to obtain the optimal nonamer composition of mRNA sequences, and applies a predictor to identify human mRNA subcellular localization. Recently, deep learning methods [44–47] have been used to predict subcellular localization with good results.

However, most existing RNA subcellular localization classifiers only solve the problem of single-label classification. In fact, a single primary RNA transcript is used to make multiple proteins [48–50]. Therefore, it is of great practical significance to expand RNA subcellular localization into multi-label classification problem. In view of the above research, there is no multi-label RNA subcellular localization dataset available for this task. According to RNALocate database, we extract multi-label classification datasets about RNA-associated subcellular localizations on various types of RNAs, and then construct subcellular localization datasets on four RNA categories (mRNAs, lncRNAs, miRNAs and snoRNAs).

In this study, we utilize different nucleotide property composition models to adequately represent important information of nucleotide sequences. In the most critical part, we achieve a major challenge is to fuse the multivariate information through multiple kernel learning[51–58], based on Hilbert-Schmidt independence criterion. The optimal combined kernel can be put into an integration support vector machine model for training a multi-label RNA subcellular localization classifier. We follow Chou’s 5-steps rule [24] to go through the following five steps: (1) construct a valid benchmark dataset to train and test the predictor; (2) utilize different nucleotide property composition models to adequately represent important information of nucleotide sequences; (3) achieve a major challenge is to fuse the multivariate information through multiple kernel learning based on Hilbert-Schmidt independence criterion, and the optimal combined kernel can be put into an integration support vector machine model for training a multi-label RNA subcellular localization classifier; (4) properly perform cross-validation tests to objectively evaluate the anticipated prediction accuracy; (5) establish multiple user-friendly web-servers for different datasets.

## Results

In this section, we compare various nucleotide representations, integration strategies and classification tools on our novel benchmark datasets.

### Evaluation measurements

Ten-fold cross-validation is a statistical technique to evaluate the performance of models in turn. Six parameters are used to analyze the performance of model [59], including Average Precision (*AP*), Accuracy (*Acc*), Coverage (*Cov*), Ranking Loss (*L*_*r*_), Hamming Loss (*L*_*h*_) and One-error (*E*_*one*_).

1a $$\begin{array}{*{20}l} Acc &= \frac{1}{|D|}\sum_{i=1}^{|D|} \left| \frac{ \hat{Y_{i}} \cap Y_{i} }{ \hat{Y_{i}} \cup Y_{i}} \right| \end{array} $$

1b $$\begin{array}{*{20}l} Cov &= \frac{1}{|D|}\sum_{i=1}^{|D|} \max_{y_{p} \in Y_{i}} \hat{r}(y_{p}) -1 \end{array} $$

1c $$\begin{array}{*{20}l} AP &= \frac{1}{|D|}\sum_{i=1}^{|D|}\frac{1}{|Y_{i}|} \sum_{y_{q} \in Y_{i}} \frac{ | \{ y_{p} | \hat{r}(y_{p}) \leq \hat{r}(y_{q}), y_{p} \in Y_{i} \} | }{ \hat{r}(y_{q})} \end{array} $$

1d $$\begin{array}{*{20}l} L_{r}\! &= \!\frac{1}{|D|}\!\sum_{i=1}^{|D|}\!\frac{| \{ (y_{p},y_{q}) | \hat{f}(y_{p}) \leq \hat{f}(y_{q}), y_{p} \in Y_{i}, y_{q} \in \bar{Y_{i}} \} |}{|Y_{i}| \times |\bar{Y_{i}}|} \end{array} $$

1e $$\begin{array}{*{20}l} L_{h} &= \frac{1}{|D|}\sum_{i=1}^{|D|}\frac{| \hat{Y_{i}} \Delta Y_{i} |}{|L|} \end{array} $$

1f $$\begin{array}{*{20}l} E_{one} &= \frac{1}{|D|}\sum_{i=1}^{|D|} | \arg\max \hat{f}(y_{p}) \notin Y_{i} | \end{array} $$

where |*D*| represents the number of samples, |*L*| represents the number of labels, $\hat {r}(y)$ indicates the rank of *y* in *Y* on the descending order, $\hat {f}(y)$ represents the score of *y* predicted by the classifier, *Y* represents the real label set, $\hat {Y}$ represents the prediction label set, $\bar {Y}$ denotes the complementary set of *Y*, *Δ* stands for the symmetric difference between two label sets.

For Coverage, Ranking Loss, Hamming Loss and One-error, the model can achieve the best performance with the smallest value. For Average Precision and Accuracy, the model can achieve the best performance with the largest value.

### Performance of different nucleotide representations

We analyze seven different nucleotide property composition representations via 10-fold cross validation. Here, we compare single-kernel feature models on four RNA subcellular localization datasets, as shown in Table 1. It can be observed that kmer achieves best performance on mRNAs (AP:0.688) and lncRNAs (AP:0.745), NAC obtains best performance on miRNAs (AP:0.785), and DNC gains best performance on snoRNAs (AP:0.793). Details are shown in Additional file 1: Table S5. Also, we compare single-kernel feature models on four human RNA subcellular localization datasets, as shown in Table 2. It can be noticed that kmer achieves best performance on mRNAs (AP:0.750), lncRNAs (AP:0.753), and snoRNAs (AP:0.817), CKSNAP obtains best performance on miRNAs (AP:0.784). Details are shown in Additional file 1: Table S6.

**Table 1:** Average Precision of seven different nucleotide representations on four RNA datasets

Models	mRNAs	lncRNAs	miRNAs	snoRNAs
**K**_kmer4_	**0.688**	**0.745**	0.782	0.782
**K**_kmer1234_	0.626	0.730	0.775	0.775
**K**_RCKmer_	0.658	0.733	0.726	0.775
**K**_NAC_	0.572	0.722	**0.785**	0.773
**K**_DNC_	0.668	0.737	0.760	**0.793**
**K**_TNC_	0.686	0.741	0.751	0.774
**K**_CKSNAP_	0.664	0.725	0.773	0.773

**Table 2:** Average Precision of seven different nucleotide representations on four human RNA datasets

Models	H_mRNAs	H_lncRNAs	H_miRNAs	H_snoRNAs
**K**_Kmer4_	0.726	**0.753**	0.764	**0.817**
**K**_Kmer1234_	**0.750**	0.739	0.768	0.815
**K**_RCKmer_	0.717	0.738	0.700	0.794
**K**_NAC_	0.722	0.729	0.772	0.796
**K**_DNC_	0.736	0.726	0.740	0.808
**K**_TNC_	0.726	0.732	0.716	0.803
**K**_CKSNAP_	0.723	0.738	**0.784**	0.800

**Fig. 1:**
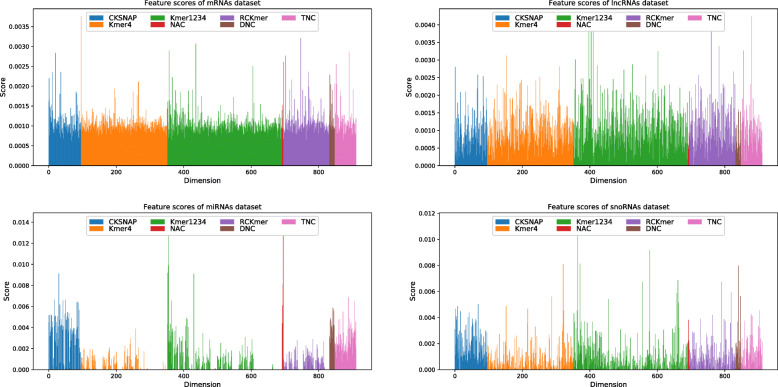
Feature importantce scores of seven characteristics on four RNA datasets

**Fig. 2:**
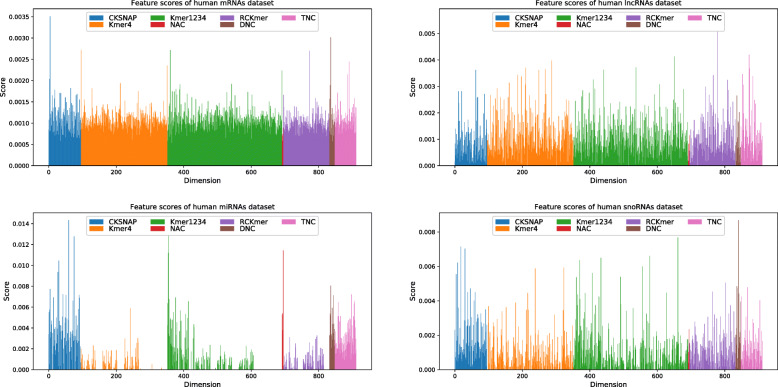
Feature importantce scores of seven characteristics on four human RNA datasets

In order to further analyze characteristics, we make use of random forest (RF) to calculate the importantce score of each feature dimension. On four RNA datasets, feature scores of mRNAs have more balanced overall distribution, but feature scores of miRNAs and snoRNAs have irregular distributions, as shown in Fig. 1. This phenomena is also reflected on four human RNA dataset, as shown in Fig. 2. It indicates that miRNAs and snoRNAs have shorter sequences with less regular nucleotide property composition information.

### Performance of different integration strategies

We study five different integration strategies with SVM model as base classifier via 10-fold cross validation, including binary relevance (BR) [59], ensemble classifier chain (ECC) [60], label powerest (LP) [59], multiple kernel learning with average weights (MK-AW), multiple kernel learning with Hilbert-Schmidt independence criterion (MK-HSIC).

Here, we compare five integrated SVM strategies on four RNA subcellular localization datasets, as shown in Table 3. It can be observed that MKSVM-HSIC achieves best performance on mRNAs (AP:0.703), lncRNAs (AP:0.757), miRNAs (AP:0.787), and snoRNAs (AP:0.800). Details are shown in Additional file 1: Table S7. Also, we compare five integrated SVM strategies on four human RNA subcellular localization datasets, as shown in Table 4. It can be observed that MK-HSIC achieves best performance on mRNAs (AP:0.755), lncRNAs (AP:0.754), miRNAs (AP:0.791), and snoRNAs (AP:0.816). Details are shown in Additional file 1: Table S8. Overall accuracy of our integration strategy is significantly higher than that of other four strategies. It can be found that multiple kernel learning has an obvious advantage over other general integration strategies in dealing with classification problems.

**Table 3:** Average Precision of five different integration strategies on four RNA datasets

Integrations	mRNAs	lncRNAs	miRNAs	snoRNAs
SVM-BR	0.651	0.737	0.724	0.775
SVM-ECC	0.671	0.735	0.725	0.775
SVM-LP	0.652	0.738	0.712	0.775
MKSVM-AW	0.699	0.755	0.784	0.792
MKSVM-HSIC	**0.703**	**0.757**	**0.787**	**0.800**

**Table 4:** Average Precision of five different integration strategies on four human RNA datasets

Integrations	H_mRNAs	H_lncRNAs	H_miRNAs	H_snoRNAs
SVM-BR	0.720	0.731	0.670	0.794
SVM-ECC	0.711	0.731	0.673	0.800
SVM-LP	0.716	0.730	0.637	0.797
MKSVM-AW ^*a*^	0.741	0.752	0.785	0.814
MKSVM-HSIC	**0.755**	**0.754**	**0.791**	**0.816**

**Fig. 3:**
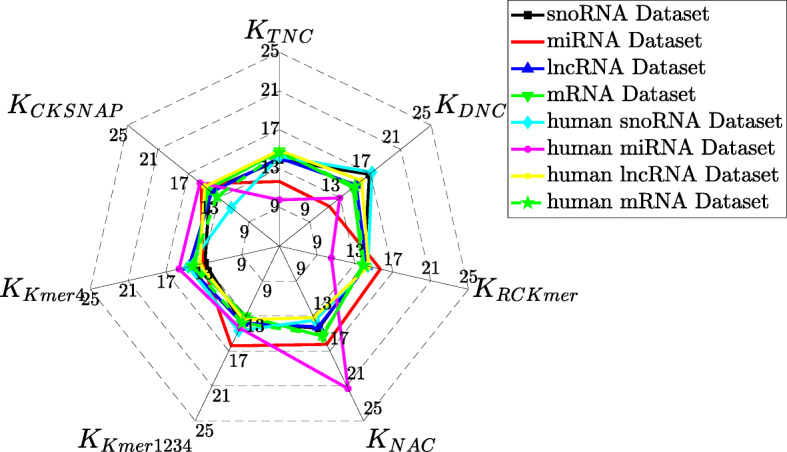
Weights for seven different kernels on various RNA datasets

According to MK-HSIC strategy, we optimize all weights of effective kernels, in order to improve the correlation between optimal combined kernel and ideal kernel. All weights for seven kernels are shown in Fig. 3. Details are shown in Additional file 1: Table S9. On miRNAs dataset, **K**_Kmer1234_ has highest kernel weight, and **K**_NAC_ has second highest kernel weight. On human miRNAs dataset, **K**_NAC_ has highest kernel weight. On other six dataset, **K**_DNC_ similarly has highest kernel weights.

### Comparison with existing classification tools

We compare the performance of different classifiers for solving multi-label classification problem via 10-fold cross validation. We use all feature sets for training SVM [61], RF [40], ML-KNN [59], extreme gradient boosting (XGBT) [62], multi-layer perceptron (MLP) [63].

Here, we compare six classification methods on four RNA subcellular localization datasets, as shown in Table 5. It can be observed that MKSVM-HSIC achieves best performance on mRNAs (AP:0.703), lncRNAs (AP:0.757) and miRNAs (AP:0.787), and XGBT obtains best performance on snoRNAs (AP:0.806). Details are shown in Additional file 1: Table S10. Also, we compare six classification methods on four human RNA subcellular localization datasets, as shown in Table 6. It can be noticed that MKSVM-HSIC achieves best performance on mRNAs (AP:0.755), lncRNAs (AP:0.754), miRNAs (AP:0.791), and snoRNAs (AP:0.816). Details are shown in Additional file 1: Table S11. As is clearly reflected by the chart, MKSVM-HSIC achieved best performance on different RNA datasets, and XGBT and RF also have good prediction results. It proves that our novel method is valid, and our new benchmark dataset is correct and meaningful.

**Table 5:** Average Precision of five different classifiers on four RNA datasets

Methods	mRNAs	lncRNAs	miRNAs	snoRNAs
SVM	0.651	0.737	0.724	0.775
RF	0.640	0.753	0.728	0.776
ML-KNN	0.576	0.683	0.673	0.748
XGBT	0.701	0.751	0.785	**0.806**
MLP	0.664	0.721	0.709	0.762
MKSVM-HSIC	**0.703**	**0.757**	**0.787**	0.800

**Table 6:** Average Precision of five different classifiers on four human RNA datasets

Methods	H_mRNAs	H_lncRNAs	H_miRNAs	H_snoRNAs
SVM	0.720	0.731	0.670	0.794
RF	0.724	0.732	0.728	**0.816**
ML-KNN	0.687	0.677	0.607	0.775
XGBT	**0.755**	0.745	**0.791**	0.810
MLP	0.711	0.719	0.707	0.794
MKSVM-HSIC	**0.755**	**0.754**	**0.791**	**0.816**

**Fig. 4:**
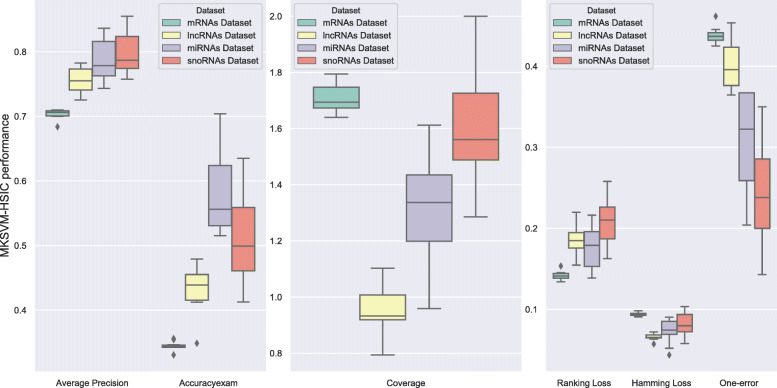
The robustness of our novel method on four RNA datasets

**Fig. 5:**
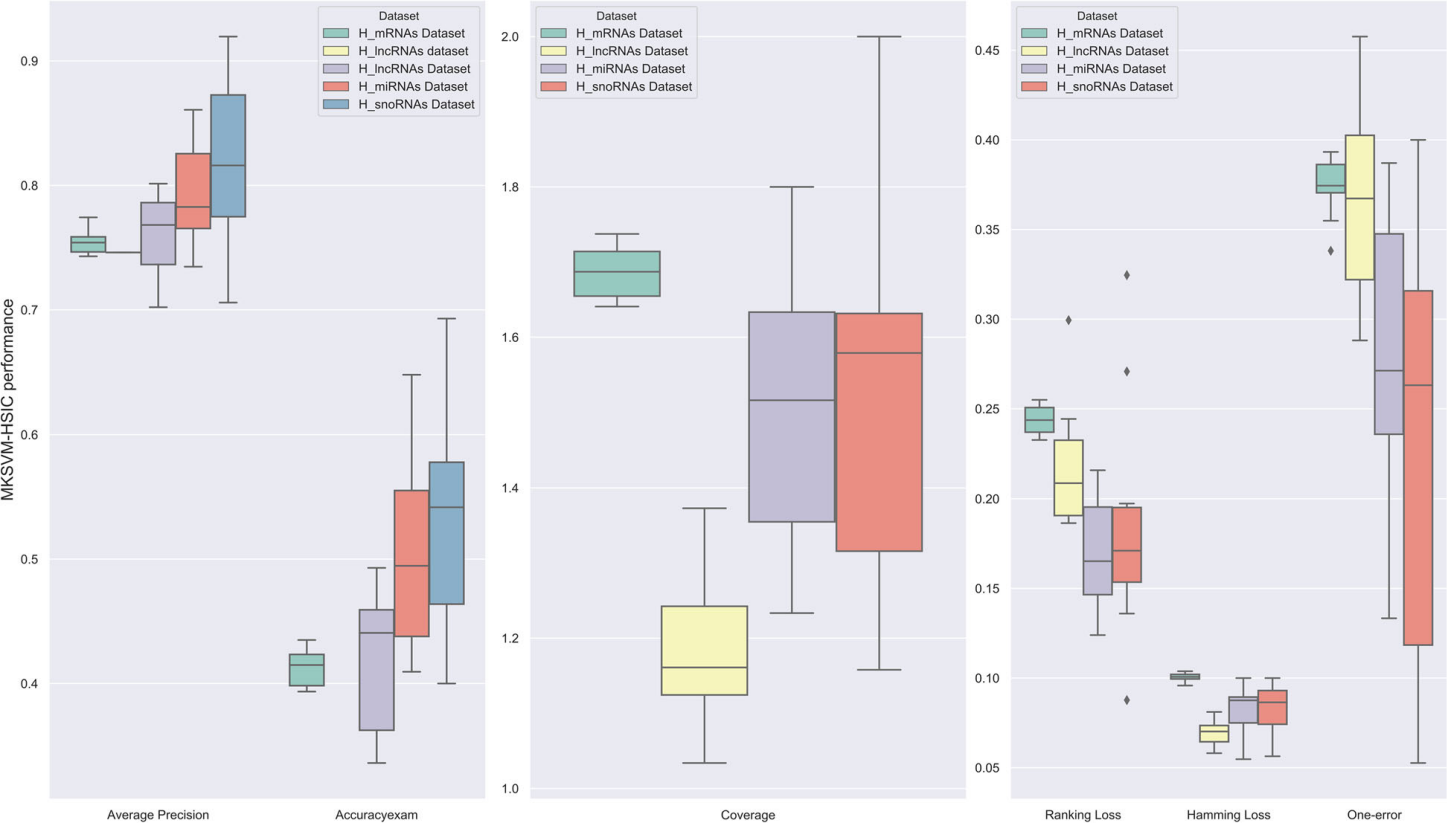
The robustness of our novel method on four human RNA datasets

In order to analyze the stability, we perform T-check on MKSVM-HSIC via 10-fold cross validation. We calculate mean value and standard deviation of Average Precision, Accuracy, Coverage, Ranking Loss, Hamming Loss and One-error, as shown in Fig. 4 on RNA dataset and Fig. 5 on human RNA dataset. It can be seen that the variance of MKSVM-HSIC is small, so the stability and robustness of our method is very excellent. Details are shown in Additional file 1: Table S12.

Importantly, RNAs are assigned in specific locations of a cell, enabling the cell to implement diverse biochemical processes in the way of concurrency. To be specific, our novel method performs outstanding rather than other prediction tools on our novel benchmark datasets. Moreover, we establish user-friendly web server with the implementation of our method.

### Web server

A web server is built for the new proposed method in this paper, the URL is http://lbci.tju.edu.cn/Online_services.htm, including four servers: LocmRNA, LocmiRNA, LocmiRNA and LocsnoRNA. Each one supports two prediction formats, an on-line input single sequence or an entire multiple sequence upload file. The sequence format must be.*fasta*. It will return the possibility of each label for RNA subcellular localization, and also give the suggested labels as final prediction result.

## Conclusion

In this paper, we establish multi-label benchmark data sets for various RNA subcellular localizations to verify prediction tools. Furthermore, we design an integration SVM prediction model with one-vs-rest strategy to fuse a variety of nucleic acid sequence to identify RNA subcellular localization. Finally, we propose user-friendly web server with the implementation of our method, which is a useful platform for research community. However, we only consider the frequency information of the sequence, and more characteristic information can be added in the future.In addition, deep learning can be introduced to solve the problem of multiple tags and multiple classifications, which may have good results.

## Methods

In this study, we establish RNA subcellular localization datasets, and then propose an integration learning model for multi-label classification. The flowchart of our method is show in Figure S1.

### Benchmark dataset

**Fig. 6:**
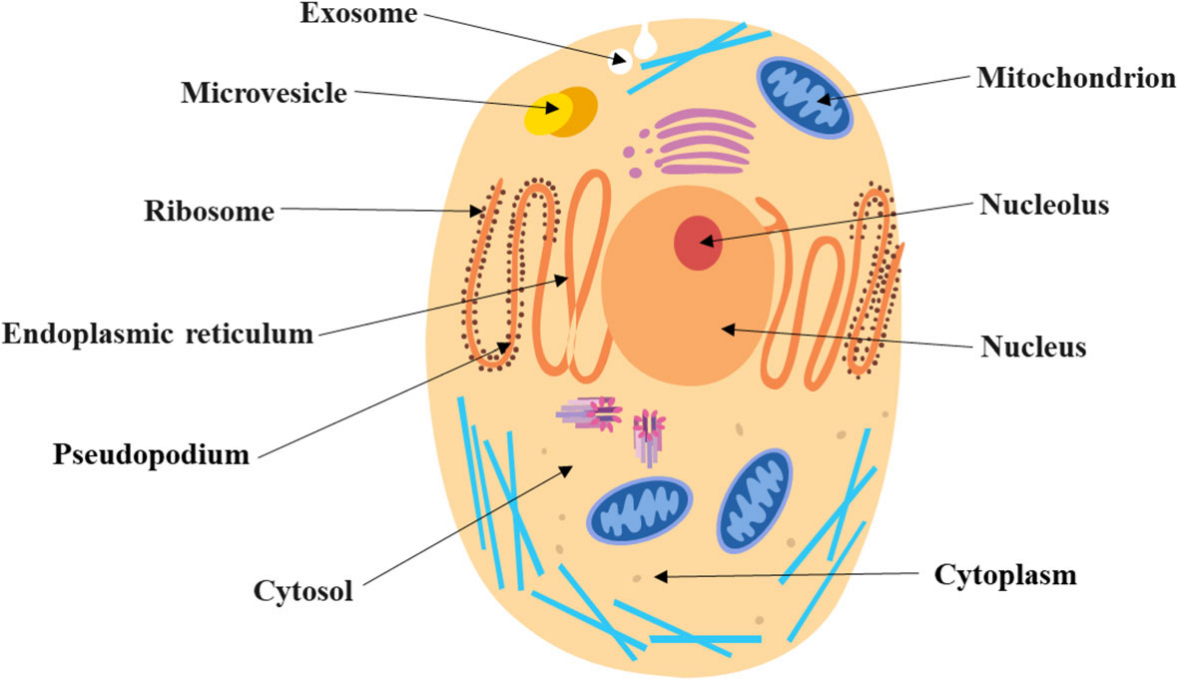
Schematic diagram of RNA subcellular localizations in cells

RNAs are generally divided into two categories. One is encoding RNAs, such as messenger RNAs (mRNAs), which play a very important role in transcription. Other is non-coding RNAs, including long non-coding RNA (lncRNA), microRNA (miRNA), small nucleolar RNA (snoRNA), which play an irreplaceable regulatory role in life. In order to study subcellular localization for Homo sapiens, we further establish human RNA subcellular localization datasets. Subcellular localizations of various RNAs in cells are shown in Fig. 6.

**Fig. 7:**
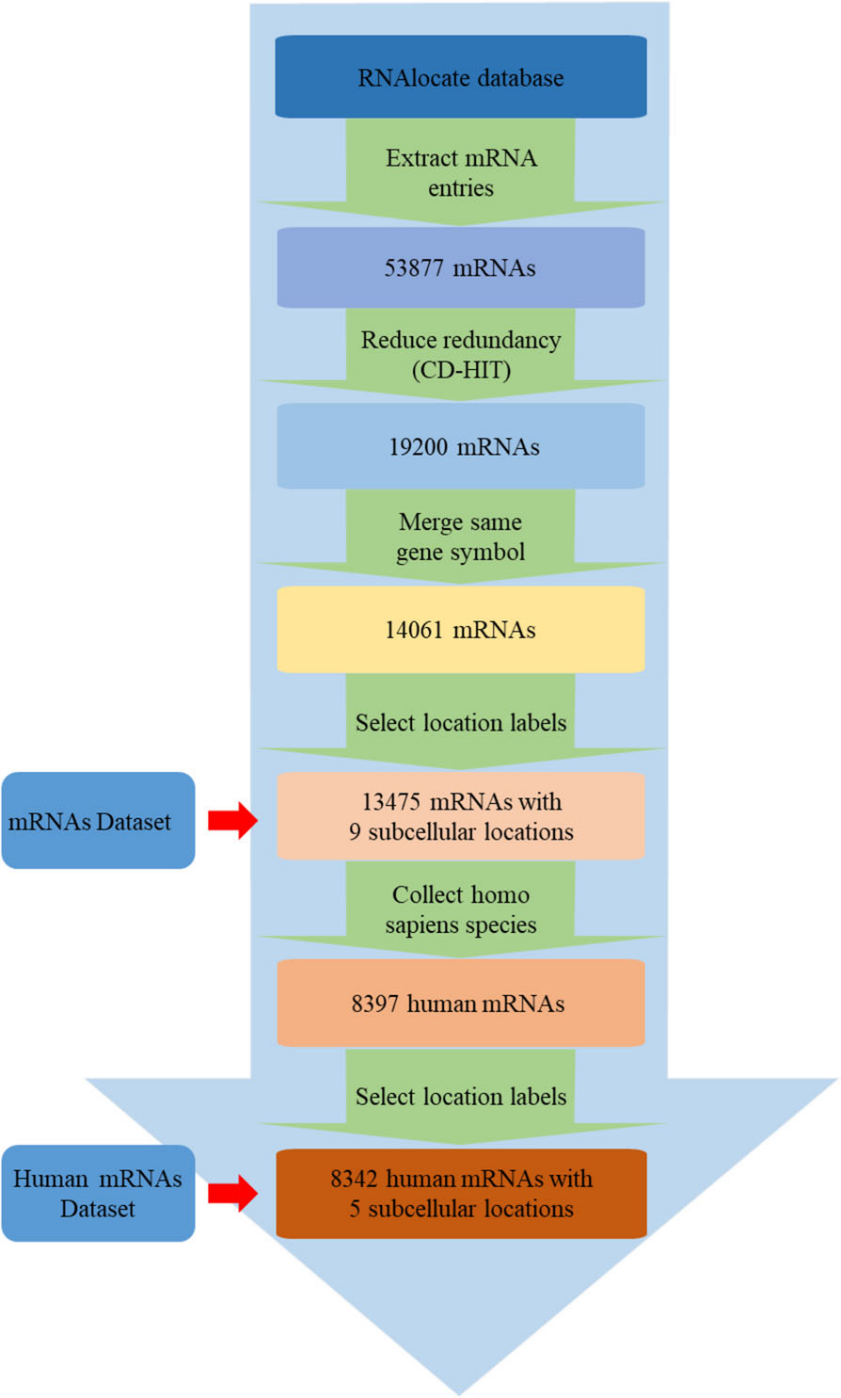
The flowchart of mRNA subcellular localization dataset construction framework

We use the database of RNA subcellular localization in order to integrate, analyze and identify RNA subcellular localization for speeding up RNA structural and functional researches. The first release of RNALocate (http://www.rna-society.org/rnalocate/) contains more than 42,000 manually engineered RNA-associated subcellular locali- zation and experimental evidence entries in more than 23100 RNA sequences, 65 organisms (e.g., homo sapiens, mus musculus, saccharomyces cerevisiae), localization of 42 subcells (e.g., cytoplasm, nucleus, endoplasmic reticulum, ribosomes), and 9 RNA categories (e.g., mRNA, microRNA, lncRNA, snoRNA). Thus, RNALocate provides a comprehensive source of subcellular localization and even insight into the function of hypothetical or new RNAs. We extract multi-label classification datasets about RNA-associated subcellular localizations on four RNA categories (mRNAs, lncRNAs, miRNAs and snoRNAs). The flowchart of mRNA subcellular localization dataset construction framework is shown in Fig. 7.

#### RNA subcellular localization datasets

We extract four RNA subcellular localization datasets, including mRNAs, lncRNAs, miRNA and snoRNAs. The procedure for constructing RNA datasets is listed as follows.

- We download total RNA entries with curated subcellular localizations from RNAlocate, and use CD-HIT [64] to remove redundant samples with a cutoff of 80%.
- We delete samples with duplicate Gene ID and remove samples without corresponding subcellular localization labels, and then construct four RNA subcellular localization datasets.
- We count the number of samples for each category of subcellular localization labels, and then select some categories with the sample size greater than a reasonable threshold (*N*/*N*_*max*_>1/30).

**Fig. 8:**
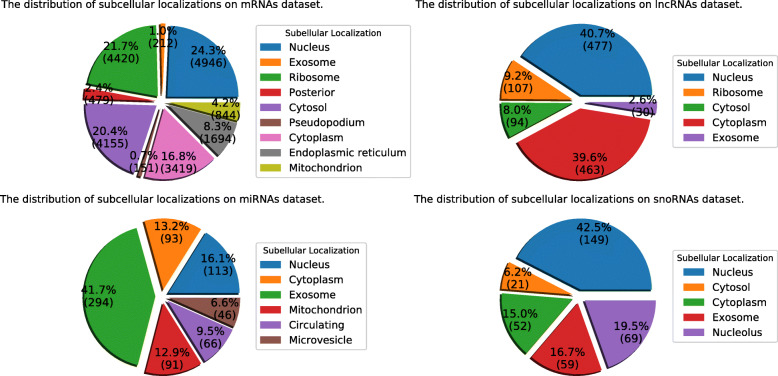
The statistical distributions of four RNA subcellular localization datasets

The statistical distributions of these four RNA datasets are shown in Fig. 8. Details are shown in Additional file 1: Table S1-S2.

#### Human RNA subcellular localization datasets

We also extract four Homo sapiens RNA subcellular localization datasets, including H_mRNAs, H_lncRNAs, H_miRNA and H_snoRNAs. The procedure for constructing human RNA datasets is listed as follows.

- We screen out samples of homo sapiens on above four RNA datasets, and construct four human RNA subcellular localization datasets.
- We count the number of samples for each category, and then select some categories with the sample size greater than a reasonable threshold (*N*/*N*_*max*_>1/12).

**Fig. 9:**
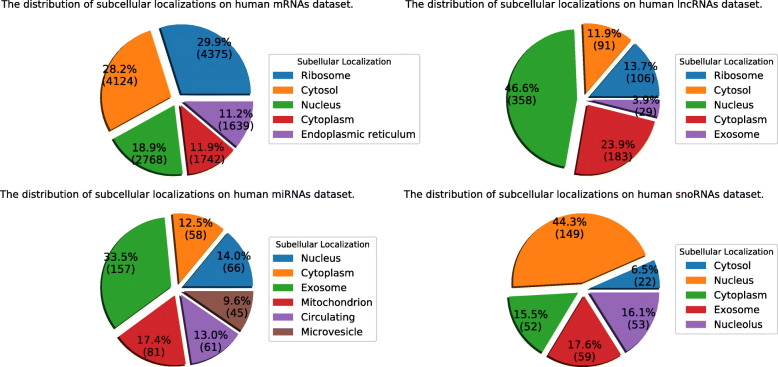
The statistical distributions of four human RNA subcellular localization datasets

The statistical distributions of these four human RNA datasets are shown in Fig. 9. Details are shown in Additional file 1: Table S3-S4.

### Nucleotide property composition representation

RNA sequence can be represented as follow: *S*=(*s*_1_,⋯,*s*_*l*_,⋯,*s*_*L*_), where *s*_*l*_ denotes the *l*-th ribonucleic acid and *L* denotes the length of *S*. How to formulate varied length RNA sequences as fixed length features, is the key point to effective operational problem-solving. Many studies have shown that the RNA sequence can be encoded by nucleotide property composition representation [65], which can profoundly affect the way of body behaves. Here, we encode the RNA sequence in order to better mine and explore information patterns.

#### k-mer nucleotide composition

For *k*-mer descriptor, RNAs are represented as occurrence frequencies of *k* neighboring nucleic acids, which has been successfully applied to human gene regulatory sequence prediction and enhancer identification. The *k*-mer (e.g. *k*=2) descriptor can be calculated as follows.

2 $$ f(t) = \frac{N(t)}{N-k+1}, \quad t \in \{AA, AC, AG, TT\}  $$

where *N*(*t*) is the number of *k*-mer type *t*, while *N* is the length of a nucleotide sequence.

For *k*=1,2,3,4, there are four combinations together, each of which has 4^*k*^ distinct types of nucleotide characteristics. Therefore, we extract 340-dimensional feature vector *F*_*kmer*1234_.

Only remaining 4-mer, there are 4^4^ types of nucleotide characteristics. Therefore, we extract 256-dimensional feature vector *F*_*kmer*4_.

#### Reverse compliment k-mer

The reverse compliment *k*-mer (RCKmer) is a variant of *k*-mer descriptor, which is not expected to be strand-specific. For instance, there are 16 types of 2-mer (‘AA’, ‘AC’, ‘AG’, ‘AT’, ‘CA’, ‘CC’, ‘CG’, ‘CT’, ‘GA’, ‘GC’, ‘GG’, ‘GT’, ‘TA’, ‘TC’, ‘TG’, ‘TT’), ‘TT’ is reverse compliment with ‘AA’. After removing the reverse compliment *k*-mer, there are only 10 distinct types of *k*-mer in the reverse compliment *k*-mer approach (‘AA’, ‘AC’, ‘AG’, ‘AT’, ‘CA’, ‘CC’, ‘CG’,‘GA’, ‘GC’, ‘TA’).

For 4-mer with 256 types, after removing reverse compliment 4-mer, there are 136 distinct types in the reverse compliment *k*-mer approach. Therefore, we extract 136-dimensional feature vector *F*_*RCKmer*_.

#### Nucleic acid composition

The nucleic acid composition (NAC) encodes the frequency of each nucleic acid type in a nucleotide sequence, which is similar to 1-mer. The frequency of each natural nucleic acid (‘A’, ‘C’, ‘G’, ‘T’ or ‘U’) can be calculated as follows.

3 $$ f(t) = \frac{N(t)}{N}, \qquad t \in \{A, C, G, T(U)\}  $$

where *N*(*t*) is the number of nucleic acid type *t*, while *N* is the length of a nucleotide sequence.

Therefore, we extract 4-dimensional feature vector *F*_*NAC*_.

#### Di-nucleotide composition

The di-nucleotide composition (DNC) encodes the frequency of each 2-tuple of nucleic acid type in a nucleotide sequence, which is similar to 2-mer. The frequency of each 2-tuple of natural nucleic acid can be calculated as follows.

4 $$ D(i,j) = \frac{N_{ij}}{N-1}, \qquad i,j \in \{A, C, G, T(U)\}  $$

where *N*_*ij*_ is the number of di-nucleotide type represented by nucleic acid types *i* and *j*.

Therefore, we extract 16-dimensional feature vector *F*_*DNC*_.

#### Tri-nucleotide composition

The tri-nucleotide composition (TNC) encodes the frequency of each 3-tuple of nucleic acid type in a nucleotide sequence, which is similar to 3-mer. The frequency of each 3-tuple of natural nucleic acid can be calculated as follows.

5 $$ D(i,j,k) = \frac{N_{ijk}}{N-2}, \qquad i,j,k \in \{A, C, G, T(U)\}  $$

where *N*_*ijk*_ is the number of di-nucleotide type represented by nucleic acid types *i*, *j* and *k*.

Therefore, we extract 64-dimensional feature vector *F*_*TNC*_.

#### Composition of k-spaced nucleic acid pair

The composition of *k*-spaced nucleic acid pair (CKSNAP) is used to calculate the frequency of nucleic acid pairs separated by any *k* nucleic acids (*k*=0,1,2,…). For each *k*-space, there are 16 types of nucleic acid pair composition (‘A...A’, ‘A...C’, ‘A...G’, ‘A...T’, ‘C...A’, ‘C...C’, ‘C...G’, ‘C...T’, ‘G...A’, ‘G...C’, ‘G...G’, ‘G...T’, ‘T...A’, ‘T...C’, ‘T...G’, ‘T...T’).

For *k*=0,1,2,3,4,5, there are six different combinations, each of which has 16 distinct types of nucleic acid pair composition. Therefore, we extract 96-dimensional feature vector *F*_*CKSNAP*_.

### Multiple kernel support vector machine classifier

We apply radial basis function (RBF) on above feature sets to construct corresponding kernels, respectively. The RBF kernel is defined as follows.

6 $$  {}K_{ij}=K(\mathbf{x}_{i},\mathbf{x}_{j}) = exp(-\gamma \Vert \mathbf{x}_{i} - \mathbf{x}_{j} \Vert^{2}), \ i,j=1,2,...,N  $$

where **x**_*i*_ and **x**_*j*_ are the feature vectors of samples *i* and *j*, *N* denotes the number of samples, and *γ* is the bandwidth of Gaussian kernel.

The kernel set with seven distinct kernels is denoted as follows.

7 $$\begin{array}{*{20}l}  \mathbf{K}&= \left \{ \mathbf{K}_{\text{kmer4}}, \mathbf{K}_{\text{kmer1234}}, \mathbf{K}_{\text{RCKmer}},\right.\\&\left.\qquad\mathbf{K}_{\text{NAC}}, \mathbf{K}_{\text{DNC}}, \mathbf{K}_{\text{TNC}}, \mathbf{K}_{\text{CKSNAP}} \right \} \end{array} $$

#### Hilbert-Schmidt Independence criterion-multiple kernel learning

We use multiple kernel learning (MKL) to figure out weights of above kernels, and then integrate them together “Multiple kernel support vector machine classifier”, “??”, and “??” sections. The optimal combinatorial kernel can be calculated as follows.

8 $$  \mathbf{K}^{*} = \sum_{p=1}^{7} \beta_{p} \mathbf{K}^{p}, \quad \mathbf{K}^{p} \in \mathbf{R}^{N \times N}  $$

The main purpose of hilbert-schmidt independence criterion (HSIC) [66] is to measure a difference in the distribution of two variables, which is similar to the covariance and is itself constructed according to the covariance. Let **X**∈**R**^*N*×*d*^ and **Y**∈**R**^*N*×1^ be two variables from a data set of $\mathbf {Z}=\left \{(\mathbf {x}_{i},y_{i})\right \}^{N}_{i=1}$, which is jointly from some probability distribution *Pr*_**x***y*_. HSIC measures the independence between **x** and *y* by calculating the norm of cross-covariance operator over domain **X**×**Y**.

Hilbert-Schmidt operator norm “Hilbert-Schmidt Independence criterion-multiple kernel learning” section of *C*_**x***y*_ is defined as follows.

9 $$ HSIC(\mathbf{F},\mathbf{G},Pr_{\mathbf{x}y}) = \Vert C_{\mathbf{x}y} \Vert^{2}_{HS}  $$

Given set **Z**, empirical estimate of HSIC is computed as follows.

10 $$ \begin{aligned}  HSIC(\mathbf{F},\mathbf{G},\mathbf{Z}) &= \frac{1}{N^{2}}tr(\mathbf{K}\mathbf{U}) - \frac{2}{N^{3}}\mathbf{e}^{T}\mathbf{K}\mathbf{U}\mathbf{e} + \frac{1}{N^{4}}\mathbf{e}^{T}\mathbf{K}\mathbf{e}\mathbf{e}^{T}\mathbf{U}\mathbf{e}\\ &= \frac{1}{N^{2}} \left [ tr(\mathbf{K}\mathbf{U}) \!- \! \frac{1}{N}tr(\mathbf{K}\mathbf{U}\mathbf{e}\mathbf{e}^{T}) - \frac{1}{N}tr(\mathbf{U}\mathbf{K}\mathbf{e}\mathbf{e}^{T}) \right. \\ & \left. + \frac{1}{N^{2}}tr(\mathbf{U}\mathbf{e}\mathbf{e}^{T}\mathbf{K}\mathbf{e}\mathbf{e}^{T}) \right ] \\ &= \frac{1}{N^{2}} tr[\mathbf{K}(\mathbf{I} - \frac{1}{N}\mathbf{e}\mathbf{e}^{T})\mathbf{U}(\mathbf{I} - \frac{1}{N}\mathbf{e}\mathbf{e}^{T})]\\ &=\frac{1}{N^{2}}tr(\mathbf{K}\mathbf{H}\mathbf{U}\mathbf{H}) \overset{\triangle}{=} HSIC(\mathbf{K},\mathbf{U}) \end{aligned}  $$

where **F** is the RKHS of feature set **X**,**G** is the RKHS of label set **Y**,**e**=(1,...,1)^*T*^∈**R**^*N*×1^,**H**=**I**−**ee**^*T*^/*N*∈**R**^*N*×*N*^ (centering matrix), **K**,**U**∈**R**^*N*×*N*^ are kernel matrices with **K**_*ij*_=*k*(**x**_*i*_,**x**_*j*_) and **U**_*ij*_=*l*(*y*_*i*_,*y*_*j*_),**I**∈**R**^*N*×*N*^ is the identity matrix. The stronger the dependence between **K** and **U**, the larger the value. **K** and **U** are independent between each other, when *HSIC*(**K**,**U**)=0.

Enligthened by HSIC [67], we define optimization function as follows.

11a $$\begin{array}{*{20}l}  \underset{\pmb{\beta},\mathbf{K}^{*}}{max} \ HSIC(\mathbf{K}^{*},\mathbf{U}) \end{array} $$

11b $$\begin{array}{*{20}l} HSIC(\mathbf{K}^{*},\mathbf{U}) = \frac{1}{N^{2}}tr(\mathbf{K}^{*}\mathbf{H}\mathbf{U}\mathbf{H}) \end{array} $$

11c $$\begin{array}{*{20}l}  subject \ to \ \mathbf{K}^{*} = \sum_{p=1}^{P} \beta_{p}\mathbf{K}^{p}, \end{array} $$

11d $$\begin{array}{*{20}l} \beta_{p} \ge 0, \ p = 1,2,...,P, \end{array} $$

11e $$\begin{array}{*{20}l} \sum_{p=1}^{P} \beta_{p} = 1 \end{array} $$

where **K**^∗^∈**R**^*N*×*N*^ is the optimal kernel of feature space, and $\mathbf {U} = \mathbf {y}_{train}\mathbf {y}_{train}^{T} \in \mathbf {R}^{N \times N}$ is ideal kernel matrix (label kernel), *β*∈**R**^*P*×1^ is the kernel weight vector. We aim to maximize HSIC between **K**^∗^ and **U**.

Convex quadratic programming problem can be solved as follows.

12a $$\begin{array}{*{20}l}  \underset{\pmb{\beta},\mathbf{K}^{*}}{min} -\frac{1}{N^{2}}tr(\mathbf{K}^{*}\mathbf{H}\mathbf{U}\mathbf{H}) + \nu_{1}\Vert \pmb{\beta} \Vert^{2} \end{array} $$

12b $$\begin{array}{*{20}l} subject \ to \ \mathbf{K}^{*} = \sum_{p=1}^{P} \beta_{p}\mathbf{K}^{p}, \end{array} $$

12c $$\begin{array}{*{20}l} \beta_{p} \ge 0, \ p = 1,2,...,P, \end{array} $$

12d $$\begin{array}{*{20}l} \sum_{p=1}^{P} \beta_{p} = 1 \end{array} $$

where *ν*_1_ is *L*_2_ norm regularization term. The final training and testing kernels are linearly weighted by *β*, respectively.

#### Support vector machine

Support vector Machine [68] was first proposed by Cortes and Vapnik [69]. It deals primarily with dichotomies. Given a dataset of instance-label pairs {**x**_*i*_,*y*_*i*_},*i*=1,2,...,*N*, the classification decision function realized by SVM is expressed as follows.

13 $$  f(\mathbf{x}) = sign[\sum_{i=1}^{N} y_{i}\alpha_{i}\cdot K(\mathbf{x},\mathbf{x}_{i})+b]  $$

where **x**_*i*_∈**R**^1×*d*^ and *y*_*i*_∈{+1,−1}.

Solving the following convex Quadratic Programming (QP) problem can obtain the coefficient *α*_*i*_.

14a $$\begin{array}{*{20}l} \text{Maximize} \quad & \sum_{i=1}^{N} \alpha_{i} - \frac{1}{2}\sum_{i=1}^{N} \sum_{j=1}^{N} \alpha_{i}\alpha_{j}\cdot y_{i}y_{j}\cdot K(\mathbf{x}_{i},\mathbf{x}_{j}) \end{array} $$

14b $$\begin{array}{*{20}l} \text{s.t.}\quad & 0 \le \alpha_{i} \le C \end{array} $$

14c $$\begin{array}{*{20}l} & \sum_{i=1}^{N} \alpha_{i}y_{i} = 0, i=1,2,...,N \end{array} $$

where *C* is a regularization parameter that controls the balance between boundary and misclassification errors, and when the corresponding *α*_*j*_>0,**x**_*j*_ is called support vector.

#### One-vs-rest strategy

We use an indirect strategy to solve multi-label classification problem, which can be solved by converting multi-label problem into multiple binary classification problems. The one-vs-rest strategy is to treat one class as positive samples and the rest classes as negative samples. We can build a binary classifier for each class label, thus construct a total of *k* binary classifiers.

## Supplementary Information

**Additional file 1** Supplemental charts for the article are in the supplemental data file and include 12 more comprehensive tables and a flowchart.

## Data Availability

A web server is built for the new proposed method in this paper, the URL is http://lbci.tju.edu.cn/Online_services.htm, including four servers: LocmRNA, LocmiRNA, LocmiRNA and LocsnoRNA.

## Abbreviations

mRNAs: message RNAs
miRNAs: micro RNAs
lncRNAs: long non-coding RNAs
snoRNAs: small nucleolar RNAs
Ap: Average Precision
Acc: Accuracy
Cov: Coverage
*L*_*r*_: Ranking Loss
*L*_*h*_: Hamming Loss
*E*_*one*_: One-error
BR: Binary Relevance
ECC: Ensemble Classifier Chain
LP: Label Powerest
MK-AW: multiple kernel learning with average weights
MK-HSIC: multiple kernel learning with Hilbert-Schmidt independence criterion
RF: Random Forest
ML-KNN: Mutil-label K-Nearest Neighbors
XGBT: extreme gradient boosting
MLP: multi-layer perceptron
RCKmer: reverse compliment k-mer
NAC: nucleic acid composition
DNC: Di-nucleotide composition
TNC: Tri-nucleotide composition
CKSNAP: composition of k-spaced nucleic acid pair
RBF: radial basis function
MKL: multiple kernel learning
HSIC: Hilbert-Schmidt independence criterion
QP: Quadratic Programming
SVM: Support vector Machine
