# Supplementary material for: Identify RNA-associated subcellular localizations based on multi-label learning using Chou’s 5-steps rule
Source: BMC Genomics. 2021 Jan 15;22:56. doi: 10.1186/s12864-020-07347-7 (PMC7811227; doi:10.1186/s12864-020-07347-7)
Supplement: Supplementary file 1 — Additional file 1 Supplemental charts for the article are in the supplemental data file and include 12 more comprehensive tables and a flowchart. [file 12864_2020_7347_MOESM1_ESM.pdf]

Supplementary Materials

Table S1: RNA subcellular localization datasets before selection.

Location	mRNA	lncRNA	miRNA	snoRNA
Nucleus	4947	477	114	150
Ribosome	4420	108	-	-
Cytosol	4155	94	-	22
Cytoplasm	3420	464	93	53
Endoplasmic reticulum	1695	7	-	-
Exosome	845	30	295	59
Mitochondrion	480	2	91	-
Posterior	212	-	-	-
Pseudopodium	152	-	-	-
Axon	99	4	5	-
Anterior	90	-	-	-
Vegetal	84	-	-	-
Perinuclear	58	1	-	-
Germ plasm	57	-	-	-
Apical	57	-	-	-
Dendrite	54	-	2	-
Peroxisome	34	-	-	-
Cell cortex	28	-	-	-
Cellular bud	27	-	-	-
Cell body	17	-	4	-
Cell leading edge	13	1	-	-
Synapse	12	1	10	-
Lamellipodium	10	1	-	-
Basal	10	-	-	-
Mitotic spindle	7	-	-	-
Cytoskeleton	7	-	-	-
Centrosome	5	-	-	-
Cell junction	4	-	-	-
Growth cone	3	-	-	-
Nucleolus	2	1	5	69
Somatodendritic compartment	2	-	-	-
Axons	2	-	-	-
Golgi apparatus	1	-	-	-
Dorsal	1	-	-	-
Endoplasmic Reticulum	1	-	-	-
Circulating	-	1	67	-
Nucleoplasm	-	1	-	-
Microvesicle	-	-	47	-
Extracellular vesicle	-	-	2	-
Chloroplast	-	-	1	-

Table S2: RNA subcellular localization datasets after selection.

Location	mRNA	lncRNA	miRNA	snoRNA
Nucleus	4947	477	114	150
Ribosome	4420	108	-	-
Cytosol	4155	94	-	22
Cytoplasm	3420	464	93	53
Endoplasmic reticulum	1695	-	-	-
Exosome	845	30	295	59
Mitochondrion	480	-	91	-
Posterior	212	-	-	-
Pseudopodium	152	-	-	-
Circulating	-	-	67	-
Microvesicle	-	-	47	-
Nucleolus	-	-	-	69
Overall locative samples	20326	1173	707	353
Overall actual samples	13,475	965	488	207

Table S3: Human RNA subcellular localization datasets before selection.

Location	H_mRNA	H_lncRNA	H_miRNA	H_snoRNA
Ribosome	4375	106	-	-
Cytosol	4124	92	-	22
Nucleus	2769	359	66	149
Cytoplasm	1743	184	59	52
Endoplasmic reticulum	1639	-	-	-
Exosome	51	30	158	59
Mitochondrion	5	-	82	-
Circulating	-	-	61	-
Microvesicle	-	-	45	-
Nucleolus	-	-	-	54
Overall	8397	588	305	191

Table S4: Human RNA subcellular localization datasets after selection.

Location	H_mRNA	H_lncRNA	H_miRNA	H_snoRNA
Ribosome	4375	106	-	-
Cytosol	4124	92	-	22
Nucleus	2769	359	66	149
Cytoplasm	1743	184	59	52
Endoplasmic reticulum	1639	-	-	-
Exosome	-	30	158	59
Mitochondrion	-	-	82	-
Circulating	-	-	61	-
Microvesicle	-	-	45	-
Nucleolus	-	-	-	54
Overall locative samples	14650	771	471	336
Overall actual samples	8342	588	305	191

Table S5: The performance of seven different nucleotide representations on four RNA datasets.

Datasets	Models	Average Precision	Accuracy	Coverage	Ranking Loss	Hamming Loss	One-error
mRNAs	$\mathbf{K^a}_{\text{Kmer4}}$	0.688	0.270	1.802	0.153	0.095	0.455
	$\mathbf{K^a}_{\text{Kmer1234}}$	0.626	0.089	2.150	0.196	0.102	0.532
	$\mathbf{K^a}_{\text{RCKmer}}$	0.658	0.288	1.943	0.170	0.103	0.499
	$\mathbf{K^a}_{\text{NAC}}$	0.572	0.013	2.439	0.228	0.106	0.605
	$\mathbf{K^a}_{\text{DNC}}$	0.668	0.250	1.934	0.168	0.098	0.476
	$\mathbf{K^a}_{\text{TNC}}$	0.686	0.296	1.824	0.155	0.096	0.457
	$\mathbf{K^a}_{\text{CKSNAP}}$	0.664	0.333	1.919	0.167	0.108	0.488
	Ave-Wt^b	0.699	**0.344**	1.730	0.144	0.096	0.444
	**MKSVM-HSIC**	**0.703**	**0.344**	**1.711**	**0.142**	**0.094**	**0.438**
lncRNAs	$\mathbf{K^a}_{\text{Kmer4}}$	0.745	0.402	0.967	0.193	0.070	0.424
	$\mathbf{K^a}_{\text{Kmer1234}}$	0.730	0.103	1.009	0.203	0.078	0.451
	$\mathbf{K^a}_{\text{RCKmer}}$	0.733	0.254	0.989	0.198	0.074	0.449
	$\mathbf{K^a}_{\text{NAC}}$	0.722	0.371	1.022	0.206	0.080	0.465
	$\mathbf{K^a}_{\text{DNC}}$	0.737	**0.434**	0.987	0.197	0.073	0.437
	$\mathbf{K^a}_{\text{TNC}}$	0.741	0.368	0.968	0.192	0.071	0.433
	$\mathbf{K^a}_{\text{CKSNAP}}$	0.725	0.354	0.997	0.199	0.074	0.465
	Ave-Wt^b	0.755	0.427	0.941	0.185	**0.066**	0.406
	**MKSVM-HSIC**	**0.757**	**0.434**	**0.934**	**0.183**	**0.066**	**0.402**
miRNAs	$\mathbf{K^a}_{\text{Kmer4}}$	0.782	0.577	1.367	0.186	0.074	0.312
	$\mathbf{K^a}_{\text{Kmer1234}}$	0.775	0.502	1.375	0.187	0.079	0.333
	$\mathbf{K^a}_{\text{RCKmer}}$	0.726	0.400	1.471	0.210	0.088	0.421
	$\mathbf{K^a}_{\text{NAC}}$	0.785	**0.582**	**1.305**	**0.175**	**0.073**	0.318
	$\mathbf{K^a}_{\text{DNC}}$	0.760	0.469	1.447	0.199	0.083	0.353
	$\mathbf{K^a}_{\text{TNC}}$	0.751	0.469	1.447	0.201	0.084	0.372
	$\mathbf{K^a}_{\text{CKSNAP}}$	0.773	0.479	1.408	0.191	0.083	0.333
	Ave-Wt^b	0.784	0.487	1.316	0.176	0.087	0.318
	**MKSVM-HSIC**	**0.787**	**0.582**	1.311	**0.175**	**0.073**	**0.310**
snoRNAs	$\mathbf{K^a}_{\text{Kmer4}}$	0.782	0.479	1.628	0.220	0.090	0.271
	$\mathbf{K^a}_{\text{Kmer1234}}$	0.775	0.478	1.715	0.234	0.090	0.275
	$\mathbf{K^a}_{\text{RCKmer}}$	0.775	0.478	1.715	0.234	0.090	0.275
	$\mathbf{K^a}_{\text{NAC}}$	0.773	0.461	1.691	0.227	0.092	0.290
	$\mathbf{K^a}_{\text{DNC}}$	0.793	**0.528**	1.652	0.223	0.085	**0.251**
	$\mathbf{K^a}_{\text{TNC}}$	0.774	0.478	1.720	0.237	0.090	0.275
	$\mathbf{K^a}_{\text{CKSNAP}}$	0.773	0.478	1.691	0.233	0.090	0.285
	Ave-Wt^b	0.792	0.478	1.628	0.214	0.089	0.261
	**MKSVM-HSIC**	**0.800**	0.515	**1.594**	**0.205**	**0.082**	**0.251**

* *a*: Results from single kernel SVM.

* *b*: Average weights-based MKSVM.

Table S6: The performance of seven different nucleotide representations on four human RNA datasets.

Datasets	Models	Average Precision	Accuracy	Coverage	Ranking Loss	Hamming Loss	One-error
H_mRNAs	$\mathbf{K^a}_{\text{Kmer4}}$	0.726	0.369	1.797	0.277	0.110	0.422
	$\mathbf{K^a}_{\text{Kmer1234}}$	0.750	0.402	1.700	0.249	0.102	0.381
	$\mathbf{K^a}_{\text{RCKmer}}$	0.717	0.360	1.835	0.288	0.113	0.440
	$\mathbf{K^a}_{\text{NAC}}$	0.722	0.344	1.855	0.298	0.113	0.427
	$\mathbf{K^a}_{\text{DNC}}$	0.736	0.373	1.804	0.278	0.107	0.398
	$\mathbf{K^a}_{\text{TNC}}$	0.726	0.378	1.806	0.282	0.115	0.422
	$\mathbf{K^a}_{\text{CKSNAP}}$	0.723	0.383	1.818	0.285	0.115	0.429
	Ave-Wt^b	0.741	0.409	1.747	0.262	0.108	0.399
	**MKSVM-HSIC**	**0.755**	**0.414**	**1.688**	**0.244**	**0.100**	**0.374**
H_lncRNAs	$\mathbf{K^a}_{\text{Kmer4}}$	0.753	0.398	1.190	0.221	0.071	**0.367**
	$\mathbf{K^a}_{\text{Kmer1234}}$	0.739	0.374	1.250	0.233	0.074	0.381
	$\mathbf{K^a}_{\text{RCKmer}}$	0.738	0.429	1.236	0.232	0.073	0.388
	$\mathbf{K^a}_{\text{NAC}}$	0.729	**0.473**	1.301	0.247	0.078	0.391
	$\mathbf{K^a}_{\text{DNC}}$	0.726	0.472	1.301	0.247	0.078	0.398
	$\mathbf{K^a}_{\text{TNC}}$	0.732	0.441	1.257	0.235	0.074	0.398
	$\mathbf{K^a}_{\text{CKSNAP}}$	0.738	0.431	1.238	0.231	0.072	0.391
	Ave-Wt^b	0.752	0.417	1.187	0.218	**0.069**	0.372
	**MKSVM-HSIC**	**0.754**	0.418	**1.180**	**0.216**	**0.069**	**0.367**
H_miRNAs	$\mathbf{K^a}_{\text{Kmer4}}$	0.764	0.373	1.541	0.186	0.098	0.349
	$\mathbf{K^a}_{\text{Kmer1234}}$	0.768	0.380	1.551	0.186	0.098	0.329
	$\mathbf{K^a}_{\text{RCKmer}}$	0.700	0.335	1.695	0.214	0.104	0.447
	$\mathbf{K^a}_{\text{NAC}}$	0.772	**0.517**	1.567	0.192	0.085	0.309
	$\mathbf{K^a}_{\text{DNC}}$	0.740	0.391	1.725	0.222	0.099	0.359
	$\mathbf{K^a}_{\text{TNC}}$	0.716	0.349	1.613	0.196	0.101	0.428
	$\mathbf{K^a}_{\text{CKSNAP}}$	0.784	0.473	1.548	0.184	0.086	0.289
	Ave-Wt^b	0.785	0.511	1.502	0.175	**0.081**	0.293
	**MKSVM-HSIC**	**0.791**	0.514	**1.462**	**0.169**	**0.081**	**0.286**
H_snoRNAs	$\mathbf{K^a}_{\text{Kmer4}}$	**0.817**	0.522	1.555	0.191	0.086	0.225
	$\mathbf{K^a}_{\text{Kmer1234}}$	0.815	0.515	1.560	0.189	0.087	0.225
	$\mathbf{K^a}_{\text{RCKmer}}$	0.794	0.516	1.754	0.234	0.086	**0.220**
	$\mathbf{K^a}_{\text{NAC}}$	0.796	0.510	1.743	0.230	0.086	0.225
	$\mathbf{K^a}_{\text{DNC}}$	0.808	0.537	1.634	0.205	**0.081**	0.230
	$\mathbf{K^a}_{\text{TNC}}$	0.803	0.516	1.707	0.220	0.086	**0.220**
	$\mathbf{K^a}_{\text{CKSNAP}}$	0.800	0.520	1.670	0.219	0.086	0.225
	Ave-Wt^b	0.814	0.516	1.571	0.193	0.086	0.230
	**MKSVM-HSIC**	0.816	**0.538**	**1.539**	**0.184**	0.082	0.236

* a: Results from single kernel SVM.
* b: Average weights-based MKSVM.

Table S7: The performance of five different integration strategies on four RNA datasets.

Datasets	Models	Average Precision	Accuracy	Coverage	Ranking Loss	Hamming Loss	One-error
mRNAs	BR(SVM)	0.651	0.118	2.003	0.178	0.100	0.503
	ECC(SVM)	0.671	**0.352**	1.826	0.157	0.102	0.493
	LP(SVM)	0.652	0.343	1.995	0.176	0.114	0.504
	Ave-Wt	0.699	0.344	1.730	0.144	0.096	0.444
	**MKSVM-HSIC**	**0.703**	0.344	**1.711**	**0.142**	**0.094**	**0.438**
lncRNAs	BR(SVM)	0.737	0.130	0.987	0.197	0.077	0.439
	ECC(SVM)	0.735	0.135	0.999	0.200	0.077	0.439
	LP(SVM)	0.738	**0.465**	0.989	0.197	0.079	0.437
	Ave-Wt	0.755	0.427	0.941	0.185	**0.066**	0.406
	**MKSVM-HSIC**	**0.757**	0.434	**0.934**	**0.183**	**0.066**	**0.402**
miRNAs	BR(SVM)	0.724	0.485	1.516	0.211	0.087	0.415
	ECC(SVM)	0.725	0.487	1.512	0.211	0.087	0.415
	LP(SVM)	0.712	0.487	1.613	0.231	0.087	0.415
	Ave-Wt	0.784	0.487	1.316	0.176	0.087	0.318
	**MKSVM-HSIC**	**0.787**	**0.582**	**1.311**	**0.175**	**0.073**	**0.310**
snoRNAs	BR(SVM)	0.775	0.478	1.715	0.234	0.090	0.275
	ECC(SVM)	0.775	0.478	1.715	0.234	0.090	0.275
	LP(SVM)	0.775	0.478	1.715	0.234	0.090	0.275
	Ave-Wt	0.792	0.478	1.628	0.214	0.089	0.261
	**MKSVM-HSIC**	**0.800**	**0.515**	**1.594**	**0.205**	**0.082**	**0.251**

Table S8: The performance of five different integration strategies on four human RNA datasets.

Datasets	Models	Average Precision	Accuracy	Coverage	Ranking Loss	Hamming Loss	One-error
H_mRNAs	BR(SVM)	0.720	0.348	1.824	0.285	0.114	0.412
	ECC(SVM)	0.711	0.350	1.873	0.306	0.116	0.447
	LP(SVM)	0.716	**0.431**	1.845	0.291	0.119	0.438
	Ave-Wt	0.741	0.409	1.747	0.262	0.108	0.399
	**MKSVM-HSIC**	**0.755**	0.414	**1.688**	**0.244**	**0.100**	**0.374**
H_lncRNAs	BR(SVM)	0.731	**0.474**	1.299	0.246	0.078	0.388
	ECC(SVM)	0.731	0.472	1.311	0.248	0.078	0.386
	LP(SVM)	0.730	**0.474**	1.301	0.247	0.078	0.389
	Ave-Wt	0.752	0.417	1.187	0.218	**0.069**	0.372
	**MKSVM-HSIC**	**0.754**	0.418	**1.180**	**0.216**	**0.069**	**0.367**
H_miRNAs	BR(SVM)	0.670	0.339	1.938	0.258	0.105	0.470
	ECC(SVM)	0.673	0.366	1.947	0.258	0.108	0.460
	LP(SVM)	0.637	0.366	2.269	0.325	0.108	0.483
	Ave-Wt	0.785	0.511	1.502	0.175	**0.081**	0.293
	**MKSVM-HSIC**	**0.791**	**0.514**	**1.462**	**0.169**	**0.081**	**0.286**
H_snoRNAs	BR(SVM)	0.794	0.516	1.754	0.234	0.086	0.220
	ECC(SVM)	0.800	0.516	1.712	0.225	0.086	0.220
	LP(SVM)	0.797	0.516	1.743	0.230	0.086	0.220
	Ave-Wt	0.814	0.516	1.571	0.193	0.086	0.230
	**MKSVM-HSIC**	**0.816**	**0.538**	**1.539**	**0.184**	**0.082**	**0.236**

Table S9: Weights for seven different kernels.

Datasets	$\mathbf{K}_{\mathrm{CKSNAP}}$	$\mathbf{K}_{\mathrm{Kmer4}}$	$\mathbf{K}_{\mathrm{Kmer1234}}$	$\mathbf{K}_{\mathrm{NAC}}$	$\mathbf{K}_{\mathrm{RCKmer}}$	$\mathbf{K}_{\mathrm{DNC}}$	$\mathbf{K}_{\mathrm{TNC}}$
mRNAs	0.1441	0.1393	0.1302	0.1532	0.1382	0.1471	0.1480
lncRNAs	0.1393	0.1457	0.1382	0.1425	0.1422	0.1513	0.1409
miRNAs	0.1529	0.1312	0.1642	0.1625	0.1567	0.1159	0.1167
snoRNAs	0.1418	0.1287	0.1382	0.1439	0.1395	0.1687	0.1392
H_mRNAs	0.1322	0.1421	0.1381	0.1518	0.1396	0.1497	0.1465
H_lncRNAs	0.1484	0.1340	0.1344	0.1318	0.1442	0.1585	0.1486
H_miRNAs	0.1547	0.1560	0.1438	0.2132	0.1050	0.1296	0.0977
H_snoRNAs	0.1134	0.1464	0.1470	0.1348	0.1436	0.1723	0.1425

Table S10: The performance of five different classifiers on four RNA datasets.

Datasets	Models	Average Precision	Accuracy	Coverage	Ranking Loss	Hamming Loss	One-error
mRNAs	SVM	0.651	0.118	2.003	0.178	0.100	0.503
	RF	0.640	0.052	2.013	0.179	0.104	0.528
	ML-KNN	0.576	0.266	2.332	0.215	0.103	0.619
	XGBT	0.701	0.272	**1.703**	**0.141**	**0.094**	0.443
	MLP	0.664	**0.353**	1.887	0.164	0.107	0.494
	Ave-Wt	0.699	0.344	1.730	0.144	0.096	0.444
	**MKSVM-HSIC**	**0.703**	0.344	1.711	0.142	**0.094**	**0.438**
lncRNAs	SVM	0.737	0.130	0.987	0.197	0.077	0.439
	RF	0.753	0.394	0.947	0.187	0.071	0.407
	ML-KNN	0.683	0.300	1.224	0.251	0.082	0.497
	XGBT	0.751	0.438	0.958	0.189	0.069	0.410
	MLP	0.721	**0.444**	1.078	0.218	0.078	0.451
	Ave-Wt	0.755	0.427	0.941	0.185	**0.066**	0.406
	**MKSVM-HSIC**	**0.757**	0.434	**0.934**	**0.183**	**0.066**	**0.402**
miRNAs	SVM	0.724	0.485	1.516	0.211	0.087	0.415
	RF	0.728	0.476	1.504	0.206	0.082	0.415
	ML-KNN	0.673	0.411	1.818	0.274	0.092	0.474
	XGBT	0.785	0.532	1.332	0.178	0.075	**0.308**
	MLP	0.709	0.442	1.586	0.232	0.098	0.441
	Ave-Wt	0.784	0.487	1.316	0.176	0.087	0.318
	**MKSVM-HSIC**	**0.787**	**0.582**	**1.311**	**0.175**	**0.073**	0.310
snoRNAs	SVM	0.775	0.478	1.715	0.234	0.090	0.275
	RF	0.776	0.482	1.686	0.229	0.089	0.275
	ML-KNN	0.748	0.467	1.831	0.266	0.094	0.324
	XGBT	**0.806**	0.479	**1.551**	**0.197**	0.091	**0.251**
	MLP	0.762	0.459	1.691	0.250	0.103	0.333
	Ave-Wt	0.792	0.478	1.628	0.214	0.089	0.261
	**MKSVM-HSIC**	0.800	**0.515**	1.594	0.205	**0.082**	**0.251**

Table S11: The performance of five different classifiers on four human RNA datasets.

Datasets	Models	Average Precision	Accuracy	Coverage	Ranking Loss	Hamming Loss	One-error
H_mRNAs	SVM	0.720	0.348	1.824	0.285	0.114	0.432
	RF	0.724	0.348	1.824	0.291	0.111	0.426
	ML-KNN	0.687	0.348	2.022	0.339	0.115	0.476
	XGBT	**0.755**	0.401	1.689	0.245	0.102	**0.374**
	MLP	0.711	0.398	1.882	0.298	0.116	0.436
	Ave-Wt	0.741	0.409	1.747	0.262	0.108	0.399
	**MKSVM-HSIC**	**0.755**	**0.414**	**1.688**	**0.244**	**0.100**	**0.374**
H_lncRNAs	SVM	0.731	**0.474**	1.299	0.246	0.078	0.388
	RF	0.732	0.420	1.277	0.239	0.074	0.389
	ML-KNN	0.677	0.389	1.531	0.295	0.079	0.459
	XGBT	0.745	0.418	1.204	0.220	0.074	0.383
	MLP	0.719	0.430	1.293	0.246	0.088	0.429
	Ave-Wt	0.752	0.417	1.187	0.218	**0.069**	0.372
	**MKSVM-HSIC**	**0.754**	0.418	**1.180**	**0.216**	**0.069**	**0.367**
H_miRNAs	SVM	0.670	0.339	1.938	0.258	0.105	0.470
	RF	0.728	0.292	1.682	0.208	0.092	0.395
	ML-KNN	0.607	0.263	2.318	0.344	0.108	0.563
	XGBT	**0.791**	0.494	1.511	0.176	**0.079**	**0.266**
	MLP	0.707	0.418	1.820	0.240	0.104	0.421
	Ave-Wt	0.785	0.511	1.502	0.175	0.081	0.293
	**MKSVM-HSIC**	**0.791**	**0.514**	**1.462**	**0.169**	0.081	0.286
H_snoRNAs	SVM	0.794	0.516	1.754	0.234	0.086	**0.220**
	RF	**0.816**	0.516	1.592	0.193	0.086	**0.220**
	ML-KNN	0.775	0.533	1.822	0.250	0.086	0.267
	XGBT	0.810	0.521	1.628	0.199	0.087	**0.220**
	MLP	0.794	0.470	1.660	0.224	0.100	0.272
	Ave-Wt	0.792	0.478	1.628	0.214	0.089	0.261
	**MKSVM-HSIC**	0.800	**0.515**	1.594	0.205	**0.082**	**0.251**

Table S12: The robustness of MKSVM-HSIC.

Datasets	Average Precision		Accuracy		Coverage		Ranking Loss		Hamming Loss		One-error	
	Mean	Std	Mean	Std	Mean	Std	Mean	Std	Mean	Std	Mean	Std
mRNAs	0.703	0.0071	0.344	0.0086	1.711	0.0508	0.142	0.0050	0.094	0.0020	0.438	0.0092
lncRNAs	0.756	0.0188	0.429	0.0362	0.945	0.0872	0.186	0.0189	0.066	0.0040	0.403	0.0321
miRNAs	0.787	0.0311	0.582	0.0648	1.311	0.1928	0.175	0.0246	0.073	0.0144	0.310	0.0586
snoRNAs	0.800	0.0349	0.515	0.0652	1.594	0.2226	0.205	0.0292	0.082	0.0139	0.251	0.0653
H_mRNAs	0.755	0.0096	0.414	0.0140	1.688	0.0354	0.244	0.0075	0.100	0.0023	0.374	0.0161
H_lncRNAs	0.754	0.0333	0.418	0.0541	1.180	0.1022	0.216	0.0332	0.069	0.0067	0.367	0.0583
H_miRNAs	0.791	0.0418	0.514	0.0791	1.462	0.1631	0.169	0.0303	0.081	0.0144	0.286	0.0805
H_snoRNAs	0.816	0.0691	0.538	0.0920	1.539	0.2654	0.184	0.0710	0.082	0.0150	0.236	0.1149

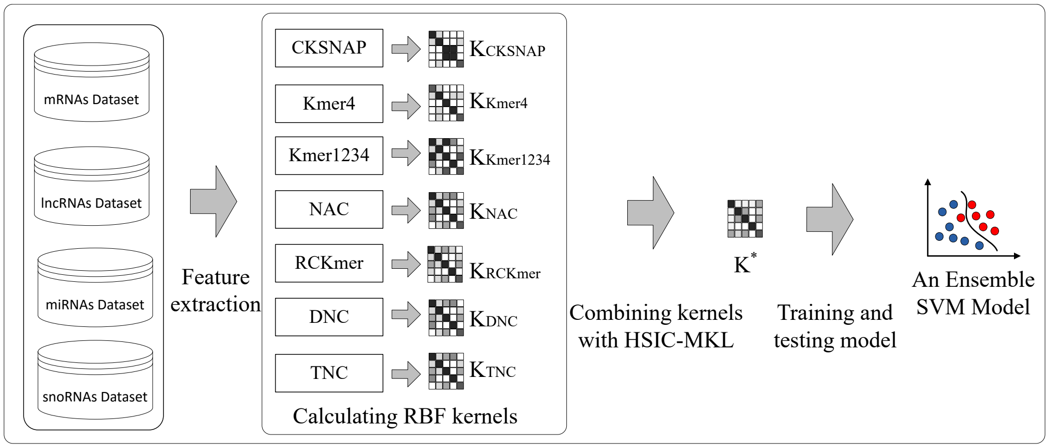

Figure S1: The flowchart of our proposed method.
